# Perioperative Outcomes of Ureteroscopy in Patients with Anatomical Anomalies of the Urinary Tract and Neurogenic Bladder: A Retrospective Comparative Study

**DOI:** 10.3390/jcm14217508

**Published:** 2025-10-23

**Authors:** Beatrice Breu, Matteo Ortolini, Audrey Masnada, François Crettenand, Nuno Grilo, Kevin Stritt

**Affiliations:** Department of Urology, University Hospital Lausanne (CHUV), University of Lausanne, 1011 Lausanne, Switzerland; beatrice.breu@chuv.ch (B.B.); matteo.ortolini@chuv.ch (M.O.); audrey.masnada@chuv.ch (A.M.); francois.crettenand@chuv.ch (F.C.); nuno.grilo@chuv.ch (N.G.)

**Keywords:** ureteroscopy, anatomical anomalies of the urinary tract, neurogenic bladder, postoperative infectious complications, kidney stones, preoperative urine culture

## Abstract

**Background/Objectives**: To compare postoperative infectious complication rates after ureteroscopy (URS) in patients with anatomical anomalies of the urinary tract and neurogenic bladder to those in patients without these conditions, and to analyze associated risk factors, urine culture profiles, and stone composition in these special populations. **Methods**: This retrospective cohort study included patients undergoing URS at a single tertiary referral center. Patients were stratified into three groups: anatomical anomalies of the urinary tract (n = 23), neurogenic bladder (n = 30), and controls (n = 297). Demographic data, perioperative details, preoperative urine culture results, stone composition, and postoperative complications were collected and compared. Univariable analyses and chi-square or Kruskal–Wallis tests were used as appropriate. **Results**: Patients with anatomical anomalies of the urinary tract and neurogenic bladder had significantly higher rates of postoperative infectious complications compared to controls (30.4% and 33.3% vs. 4.0%; *p* < 0.001). Preoperative urine cultures were more frequently positive in these groups, with *Escherichia coli*, *Enterococcus faecalis*, and *Pseudomonas aeruginosa* being the most common pathogens. Stone analysis revealed a higher proportion of struvite and mixed calcium phosphate stones in the anatomical anomalies of the urinary tract and neurogenic bladder groups compared to controls, who predominantly formed calcium oxalate stones (75%). **Conclusions**: Patients with anatomical anomalies of the urinary tract or neurogenic bladder are at higher risk of postoperative infectious complications following URS, likely due to more frequent colonization and anatomical or functional urinary tract abnormalities. These findings underscore the importance of tailored perioperative management, including targeted antimicrobial strategies and careful surgical planning, to mitigate risks in these high-risk populations.

## 1. Introduction

Ureteroscopy (URS) has become an increasingly preferred, minimally invasive approach for managing urolithiasis and upper urinary tract pathology [1,2]. Continuous advancements in endoscopic technology, laser lithotripsy, and surgical training have led to higher stone-free rates and reduced perioperative morbidity [3,4]. Despite these improvements, URS is still associated with notable risks of complications, especially infectious ones such as febrile urinary tract infections (UTIs), sepsis, or unplanned hospital readmissions, which may occur in up to 7% of cases [5,6,7]. Recent systematic reviews confirm that prolonged preoperative stenting, positive urine cultures, longer operative times, and higher comorbidity burden are independent predictors of post-URS urosepsis [7].

Patients with complex urological conditions such as congenital or acquired anatomical anomalies of the urinary tract—including ureteropelvic junction (UPJ) obstruction, ureteral strictures, or renal transplants—may have impaired urine drainage, predisposing them to residual stones and infection [8,9,10]. More recently, emerging imaging biomarkers—for example, renal pelvis urine density on CT—have been proposed as predictors of severe post-URS infectious events. Together, these observations underscore the multifactorial interplay between urinary tract anatomy, stone burden, and infection risk [11].

Similarly, neurogenic bladder dysfunction, often secondary to spinal cord injury, neurodegenerative diseases, or severe diabetic neuropathy, can result in impaired bladder emptying, chronic catheterization, and persistent bacterial colonization with multidrug-resistant organisms, all of which are known to increase postoperative UTI and sepsis risk [12,13,14]. Indeed, catheter-dependent patients with neurogenic bladder demonstrate some of the highest infection risks after endourological procedures, due to biofilm formation and challenges with complete urine sterilization [15]. Previous studies have highlighted the challenges of stone management in these patients but dedicated data focusing on post-URS infectious outcomes in these subgroups remain scarce [16].

Considering this, detailed evaluation of risk factors and outcomes in these high-risk populations is essential for clinicians to optimize patient counseling, perioperative antibiotic prophylaxis, surgical planning, and follow-up care [17]. By comparing patients with anatomical anomalies of the urinary tract or neurogenic bladder to a control population undergoing URS, this study seeks to contribute new insights that may inform more personalized perioperative care and risk mitigation strategies.

## 2. Materials and Methods

We conducted a retrospective, single-center cohort study in the Department of Urology at Lausanne University Hospital (CHUV), Switzerland. The study included all adult patients who underwent ureteroscopy (URS) for urolithiasis between 1 January and 31 December 2023. Data were extracted from the hospital’s secured electronic medical records system (Soarian^®^ Clinicals, Cerner Corporation, Kansas City, MO, USA), which routinely captures demographic, clinical, laboratory, imaging, and perioperative data. No additional data or biological samples were collected for this study.

The study population consisted of patients who underwent URS and had a documented diagnosis of either anatomical anomalies of the urinary tract or neurogenic bladder, as well as a control group of patients without known urinary tract anomalies or neurogenic dysfunction. Only patients with complete medical records documenting preoperative urine culture results, duration of JJ stenting, intraoperative parameters, and postoperative follow-up were included. A total of 15 patients were excluded: 4 due to incomplete records, 10 due to missing preoperative urine culture data, and 1 who declined institutional general consent for research use. Additionally, deceased individuals at the time of data collection were not included.

Data collected included demographics (age, sex, body mass index [BMI], ASA score, serum creatinine), stone characteristics (composition), preoperative urine culture results, details of any indwelling JJ stent, operative time, and perioperative outcomes. Specific anatomical anomalies of the urinary tract types and neurogenic bladder etiologies were categorized as described. Complications were assessed up to 30 days postoperatively.

The primary outcome was postoperative infectious complications, defined as fever > 38 °C, systemic inflammatory response, prolonged hospitalization (>3 days), or readmission for urinary tract infection. Secondary outcomes included the distribution of stone composition and preoperative urine culture pathogens across groups.

Descriptive statistics summarized patient characteristics, microbiological data, and complication rates. Group comparisons (anatomical anomalies of the urinary tract, neurogenic bladder, control) were performed using chi-square tests for categorical variables and Kruskal–Wallis tests for continuous variables. Where appropriate, post hoc pairwise comparisons were conducted. To identify independent predictors of postoperative infectious complications, a multivariable logistic regression model was constructed, adjusting for age, sex, ASA score ≥ 3, urinary catheter use, and preoperative urine culture status. All analyses were performed using Stata version 17.0 (StataCorp LLC, College Station, TX, USA). A two-sided *p*-value < 0.05 was considered statistically significant.

This retrospective study used pseudonymized data in accordance with the institutional general consent framework at Lausanne University Hospital (CHUV). As all analyses were performed on de-identified routine clinical data, additional approval from the ethics committee was not required under applicable local regulations. No data was used for individuals who explicitly refused the reuse of their medical information. During the preparation of this manuscript, the authors used ChatGPT (OpenAI, GPT-5, 2025) to assist with language refinement and editing for clarity and style. The authors reviewed and edited all generated content to ensure accuracy and scientific integrity and take full responsibility for the final content of this publication.

## 3. Results

A total of 350 patients who underwent ureteroscopy (URS) between 1 January and 31 December 2023, were included. Among these, 23 patients (6.5%) had anatomical anomalies of the urinary tract, 30 patients (8.5%) had a neurogenic bladder, and 297 patients (85%) served as the control group without known urinary tract anomalies or neurogenic bladder. Among patients with anatomical anomalies of the urinary tract, ureteral stenosis or UPJ obstruction was most common (52.2%), followed by anatomical anomalies, post-cystectomy urinary diversion, renal transplant, reflux, and other conditions (Table 1). In the neurogenic bladder group, cerebrovascular and neurodegenerative diseases were the leading causes (each 26.7%), and many patients had mixed lower urinary tract symptoms and nearly half (43.3%) required a urinary catheter (Table 2).

**Figure 1:**
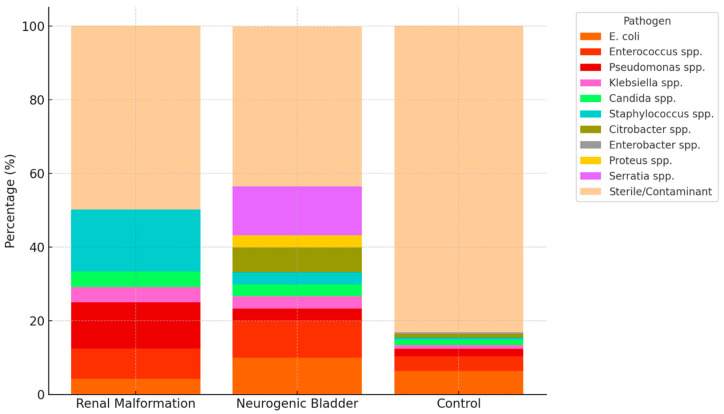
Sterile and contaminant flora were grouped together as ‘Sterile/Contaminant’ for clarity. Percentages are shown relative to each group total. The figure highlights the markedly higher prevalence of positive cultures in patients with neurogenic bladder and anatomical anomalies of the urinary tract compared to controls, with distinct microbial patterns across subgroups.

Preoperative urine cultures showed notable differences between groups (Table 3 and Figure 1). Patients with neurogenic bladder had the highest rate of positive cultures, with frequent detection of *Escherichia coli* (10%) and *Enterococcus faecalis* (10%). In the anatomical anomalies of the urinary tract group, *Pseudomonas aeruginosa* (12.5%) and *Staphylococcus aureus/lugdunensis* (16.7%) were relatively common. In contrast, the control group had a higher proportion of sterile or contaminant cultures (83.2%) compared to the anatomical anomalies of the urinary tract (50%) and neurogenic bladder (43.3%) subgroups.

Patients with anatomical anomalies of the urinary tract and neurogenic bladder differed significantly from controls in several baseline and perioperative characteristics (Table 4). Median age was higher in the anatomical anomalies of the urinary tract (67 years) and neurogenic bladder (68.5 years) groups compared to controls (54 years; *p* < 0.001). The neurogenic bladder group had a higher proportion of male patients (90%) than the anatomical anomalies of the urinary tract (52.2%) and control (69%) groups (*p* = 0.01). ASA score ≥3 was more frequent among patients with anatomical anomalies of the urinary tract (34.8%) and neurogenic bladder (76.7%) compared to controls (19.5%; *p* < 0.001). Preoperative positive urine cultures were more common in the anatomical anomalies of the urinary tract (52.2%) and neurogenic bladder (56.7%) groups than in controls (11.5%; *p* < 0.001). In the neurogenic bladder group, patients with urinary catheter (n = 13) show all, except for two, preoperative positive urinary cultures (85%). Median JJ stent dwell time and operative time were comparable across groups. Notably, infectious complications occurred more frequently in patients with anatomical anomalies of the urinary tract (30.4%) and neurogenic bladder (33.3%) than in controls (4%; *p* < 0.001).

In multivariable logistic regression analysis adjusting for age, sex, ASA score ≥ 3, urinary catheter use, and preoperative urine culture status, both anatomical anomalies of the urinary tract (OR ≈ 2.8, *p* = 0.025) and neurogenic bladder (OR ≈ 13.3, *p* < 0.001) remained independent predictors of postoperative infectious complications. Urinary catheter use was also strongly associated with infection risk (OR ≈ 17.1, *p* < 0.001). In contrast, age, sex, ASA score, and preoperative urine culture status were not independently predictive (Table 5).

**Figure 2:**
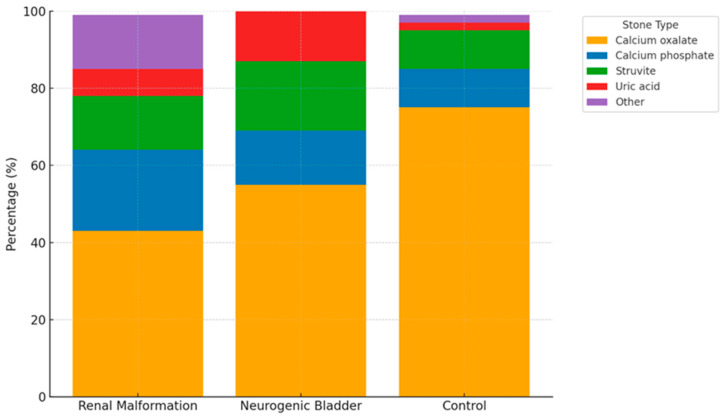
Distribution of kidney stone composition across patients with anatomical anomalies of the urinary tract, neurogenic bladder, and control group. Data are expressed as percentage of stones in each group. The ‘Other’ category includes mixed or rare stone types not otherwise classified. The figure demonstrates the higher frequency of infection-related stones (struvite and calcium phosphate) in patients with anatomical anomalies of the urinary tract and neurogenic bladder, compared to the predominance of calcium oxalate stones in the control group.

Stone composition varied between groups (Table 6, Figure 2). Calcium oxalate was the most common stone type in all groups but less frequent in patients with anatomical anomalies of the urinary tract (43%) and neurogenic bladder (55%) than in controls (75%). Calcium phosphate and struvite stones were more common in the anatomical anomalies of the urinary tract and neurogenic bladder groups than in controls. Uric acid stones were also slightly more frequent in the neurogenic bladder group compared to controls.

## 4. Discussion

In this retrospective study, we compared perioperative and postoperative outcomes of URS in patients with anatomical anomalies of the urinary tract or neurogenic bladder to a large control group. Our findings demonstrate that both anatomical anomalies of the urinary tract and neurogenic bladder are associated with distinct patient profiles and a higher risk of infectious complications after URS.

Patients with anatomical anomalies of the urinary tract presented with conditions such as UPJ obstruction or urinary diversions, which are known to complicate urinary drainage and stone management [18,19]. Anatomical abnormalities such as congenital malformations may result in impaired urinary drainage or stasis, thereby creating favorable conditions for stone formation. Similarly, the neurogenic bladder cohort included patients with cerebrovascular disease, neurodegenerative conditions, and spinal cord injuries-conditions that often impair bladder emptying and increase bacterial colonization risk [12,20].

Consistent with previous studies, we observed higher rates of positive preoperative urine cultures in patients with neurogenic bladder compared to controls [21]. Neurogenic bladder dysfunction has been widely reported as a risk factor for recurrent urinary tract infections (UTIs) and urosepsis, particularly when catheterization is required [22,23]. In our cohort, 85% of catheterized neurogenic bladder patients had positive preoperative urine cultures, confirming the strong link between catheter use and bacterial colonization. This highlights catheterization not only as a marker of disease severity but also as an independent driver of infection risk that warrants careful perioperative consideration.

Notably, patients with anatomical anomalies of the urinary tract and neurogenic bladder showed a significantly higher rate of postoperative infectious complications (30–33%) compared to only 4% in the control group. This aligns with previous reports indicating that anatomical abnormalities, impaired drainage, and colonization can increase the risk of postoperative sepsis after endourological procedures [24,25]. From a clinical perspective, these findings support tailored pathways for high-risk patients, including systematic preoperative urine cultures, extended or targeted antibiotic regimens, and staged procedures in complex cases. The routine use of ureteral access sheaths and strict monitoring of intrarenal pressures may further mitigate infectious risks [26].

The American Urological Association (AUA) recommends antibiotic prophylaxis for all patients undergoing ureteroscopy [27]. In contrast, the European Association of Urology (EAU) recommends prophylactic antibiotics only in selected cases—such as URS for proximal or impacted stones and percutaneous stone removal. For uncomplicated distal ureteral stones, antibiotic prophylaxis is not recommended, provided that the patient has a negative preoperative urine culture and no identifiable risk factors for infection [28]. Despite general agreement on the need to sterilize the urine prior to ureteroscopy in patients with positive urine cultures, the optimal duration and regimen of preoperative antibiotic therapy remain subjects of ongoing debate, with no established consensus to date.

While speculative approaches such as aminoglycoside–mannitol combinations show promise in preventing biofilm-associated infections, these remain investigational. To avoid overstating their clinical readiness, we clearly separate such emerging concepts from evidence-based strategies (e.g., strict urine sterilization, perioperative prophylaxis, minimizing operative time, and catheter management), which currently represent the most reliable means of infection prevention [29].

Our study also highlights differences in stone composition. Struvite and calcium phosphate stones were more common in patients with anatomical anomalies of the urinary tract and neurogenic bladder, reflecting the known association between infection stones and chronic bacteriuria [30,31]. Struvite stones in the urinary tract are also present in the control group, not only in patients with neurogenic disorders, and may be associated with inadequate hydration. In contrast, calcium oxalate stones predominated in the general URS population, consistent with global trends in nephrolithiasis.

These findings have practical implications. First, they underscore the need for individualized perioperative management for patients with complex urinary tract conditions. Preoperative urine cultures and targeted antibiotic regimens should be routine. Second, high-risk patients such as those with neurogenic bladder and catheter use may benefit from prolonged antibiotic therapy and closer postoperative monitoring. Third, minimizing operative times, maintaining low intrarenal pressures, and considering staged procedures when stone burden is extensive represent pragmatic steps to reduce infection-related morbidity.

Importantly, our multivariable logistic regression analysis confirmed that both anatomical anomalies of the urinary tract and neurogenic bladder remained independent predictors of infectious complications, even after adjusting for potential confounders such as age, sex, ASA score, urinary catheter use, and preoperative urine culture status. Urinary catheter use itself showed a particularly strong association with infection risk, underscoring its role as a key driver of postoperative morbidity in neurogenic bladder patients. This finding emphasizes the need for tailored catheter management strategies and highlights an area for targeted intervention in future clinical protocols.

This study has several limitations that should be acknowledged. First, its retrospective design and single-center setting may limit the generalizability of the findings to other institutions with different patient populations or surgical protocols. The retrospective nature of the study may also have introduced selection bias, as only patients with complete and accessible records were included, potentially excluding those with incomplete documentation. Second, data collection was reliant on the accuracy and completeness of existing medical records, which may have introduced information bias or missing variables. Not all patients had complete stone composition analyses or detailed microbiological cultures, which could have led to underreporting of certain stone types or less common pathogens. Third, due to the observational design, we could not control for all potential confounding factors such as variations in perioperative antibiotic regimens, surgeon experience, or individual comorbidities beyond those captured by the ASA score. Fourth, we did not assess long-term outcomes such as stone-free rates or recurrent infections, which are relevant for understanding the broader clinical impact of ureteroscopy in these populations. Fifth, variability in surgical technique and operator experience may also have influenced perioperative outcomes, although all procedures were performed within the same tertiary referral center. Finally, the relatively small sample sizes in the anatomical anomalies of the urinary tract and neurogenic bladder subgroups limit the statistical power to detect more nuanced differences, especially for rare outcomes. Despite these limitations, the consistent trends observed strengthen the validity of our findings and underscore the need for prospective multicenter studies to confirm and expand upon these results.

This retrospective cohort study demonstrates that patients with anatomical anomalies of the urinary tract and neurogenic bladder undergoing ureteroscopy face significantly higher rates of preoperative urinary tract colonization and postoperative infectious complications compared to patients without anatomical or functional urinary tract anomalies. By quantifying infection risk and identifying independent predictors such as catheter use, our study contributes actionable evidence to guide tailored perioperative management. Future prospective studies are warranted to refine risk stratification and develop evidence-based protocols that reduce infection risk and improve outcomes for these complex patient populations.

## Figures and Tables

**Table 1:** Anatomical anomalies of the urinary tract characteristics.

Anatomical Anomalies of the Urinary Tract	Total (n = 23)
Ureteral stenosis/UPJ obstruction–no. (%)	12 (52.2)
Anatomic/malposition–no. (%)	3 (13.0)
Post-cystectomie/urinary diversion–no. (%)	3 (13.0)
Renal transplant	2 (8.7)
Reflux–no. (%)	1 (4.3)
Other–(%)	2 (8.7)

UPJ: Ureteropelvic junction. Percentages are calculated based on the total number of patients with anatomical anomalies of the urinary tract (n = 23).

**Table 2:** Neurogenic bladder characteristics.

Neurogenic Bladder	Total (n = 30)
Cerebrovascular disease (Stroke)–no. (%)	8 (26.7)
Neurodegenerative disease–no. (%)	8 (26.7)
Spinal cord injury/paraplegia–no. (%)	4 (13.3)
Diabetic or toxic neuropathy–no. (%)	3 (10.0)
Congenital disorder–no. (%)	2 (3.7)
Other–no. (%)	5 (16.7)
Type of LUTS	
Urinary retention–no. (%)	4 (13.3)
Overactive bladder/incontinence–no. (%)	7 (23.3)
Mixed symptoms–no. (%)	6 (20.0)
Use of urinary catheter–no. (%)	13 (43.3)

Percentages are calculated based on the total number of patients with neurogenic bladder (n = 30). Patients could present with multiple underlying etiologies and symptoms, which explains why the cumulative percentages exceed 100%. LUTS: lower urinary tract symptoms.

**Table 3:** Distribution of Preoperative Urine Culture Pathogens by Subgroup.

	Anatomical Anomalies of the Urinary Tract (n = 23)	Neurogenic Bladder (n = 30)	Control (n = 202)
Pathogens			
*Escherichia coli*	1 (4.2)	3 (10.0)	13 (6.4)
*Enterococcus faecalis*	2 (8.3)	3 (10.0)	8 (4.0)
*Pseudomonas aeruginosa*	3 (12.5)	1 (3.3)	4 (2.0)
*Klebsiella pneumoniae*	1 (4.2)	1 (3.3)	2 (1.0)
*Candida albicans/glabrata*	1 (4.2)	1 (3.3)	3 (1.5)
*Staphylococcus aureus/lugdunensis*	4 (16.7)	1 (3.3)	1 (0.5)
*Citrobacter freundii/koseri*	-	2 (6.7)	2 (1.0)
*Enterobacter cloacae*	-	-	1 (0.5)
*Proteus mirabilis*	-	1 (3.3)	-
*Serratia marcescens*	-	4 (13.3)	-
*Sterile/Contaminant flora*	11 (50.0)	13 (43.3)	168 (83.2)

Sterile/contaminant flora includes negative cultures and samples reported as contaminated (e.g., mixed growth without a predominant pathogen) by the microbiology laboratory. Percentages are calculated within each group.

**Table 4:** Comparison of Demographic, Perioperative, and Infectious Outcomes Among Patients with anatomical anomalies of the urinary tract, Neurogenic Bladder, and Controls Undergoing URS.

	Renal Malformation (n = 23)	Neurogenic Bladder (n = 30)	Control (n = 297)	*p* Values
Demographic				
Median age–yr (IQR)	67.0 (20–86)	68.5 (29–86)	54.0 (11–96)	<0.001
Sex, male–no (%)	12 (52.2)	27 (90.0)	205 (69.0)	0.01
Sex, female–no (%)	11 (47.8)	3 (10.0)	92 (31.0)	-
Median BMI–kg/m^2^ (IQR)	24.9 (18–36)	25.5 (18–62)	27.1 (16–52)	0.064
ASA score ≥ 3–no (%)	8 (34.8)	23 (76.7)	58 (19.5)	<0.001
Median Creatinine-mmol/l (IQR)	95 (50–364)	84 (34–183)	86 (39–348)	0.938
Intervention Details				
Positive urine culture–no (%)	12 (52.2)	17 (56.7)	34 (11.5)	<0.001
Indications for URS				
Urolithiasis–no (%)	21 (91.3)	28 (93.3)	274 (92.3)	0.353
Urogenital cancer–no (%)	2 (8.7)	2 (6.7)	23 (7.7)	-
JJ stent in situ–no (%)	14 (60.9)	27 (90.0)	248 (83.5)	0.012
Median JJ dwell time–day (IQR)	28.0 (8–65)	36.0 (6–133)	32.0 (2–365)	0.591
Median operative time–min (IQR)	48.0 (9–113)	52.0 (6–83)	43.0 (7–138)	0.789
Infectious complication				
Infectious complication–no (%)	7 (30.4)	10 (33.3)	12 (4.0)	<0.001

Data are presented as median (interquartile range) or number (percentage). *p*-values were calculated using the Kruskal–Wallis test for continuous variables and Chi-square for categorical variables, as appropriate. JJ: double-J stent; URS: ureteroscopy; ASA: American Society of Anesthesiologists score.

**Table 5:** Multivariable Logistic Regression Analysis of Predictors of Infectious Complications After URS.

	Odds Ratio (OR)	95% CI	*p*-Value
Variable			
Age (per year)	0.98	0.96–1.01	0.159
Sex (male vs. female)	0.49	0.13–1.26	0.488
ASA score ≥ 3	1.36	0.40–3.82	0.634
Urinary catheter	13.3	4.1–17.0	**<0.001**
Positive urine culture	0.50	0.26–0.87	0.008

Odds ratios (OR) are presented with 95% confidence intervals (CI). A *p*-value <0.05 was considered statistically significant. ASA: American Society of Anesthesiologists score.

**Table 6:** Comparison of Kidney Stone Composition Across Patient Groups.

	Anatomical Anomalies of the Urinary Tract (n = 14)	Neurogenic Bladder (n = 22)	Control (n = 290)
Kidney Stone Composition			
Calcium oxalate–no (%)	6 (43)	12 (55)	220 (75)
Calcium phosphate–no (%)	3 (21)	3 (14)	30 (10)
Struvite–no (%)	2 (14)	4 (18)	30 (10)
Uric acid–no (%)	1 (7)	3 (14)	5 (2)
Other–no (%)	2 (14)	0	5 (2)

Values are presented as number (%) of patients with at least one identified stone type. Patients with mixed stones may contribute to more than one category. Calcium oxalate includes monohydrate and dihydrate forms. Calcium phosphate includes carbapatite, brushite, and other phosphate subtypes. “Other” includes rare types such as cystine or proteinaceous calculi.

## Data Availability

The data presented in this study are not publicly available due to ethical and legal restrictions under institutional data protection policies. However, anonymized data may be available from the corresponding author upon reasonable request and with approval from the institutional data protection officer of the University Hospital Lausanne (CHUV).

## Abbreviations

The following abbreviations are used in this manuscript:

AUA	American Urological Association
ASA	American Society of Anesthesiologists
CHUV	Centre Hospitalier Universitaire Vaudois
CT	Computed tomography
EAU	European Association of Urology
JJ	Double J ureteral stent
OR	Odds Ratio
SFR	Stone Free Rate
URS	Ureteroscopy
UTI	Urinary Tract Infection
UPJ	Ureteropelvic Junction
